# Supplementary material for: Perioperative Glial Fibrillary Acidic Protein Is Associated with Long-Term Neurodevelopment Outcome of Infants with Congenital Heart Disease
Source: Children (Basel). 2021 Jul 29;8(8):655. doi: 10.3390/children8080655 (PMC8391328; doi:10.3390/children8080655)
Supplement: Supplementary file 1 [file children-08-00655-s001.zip › File S1_GFAP and Neurodevelopment code.pdf]

GFAP and Neurodevelopment

Luca Vedovelli

28/05/2021

Libraries

```
knitr::opts_chunk$set(
    eval = FALSE,
    message = FALSE,
    warning = FALSE,
    include = TRUE
)
```

```
library(readr)
library(tidyverse)
library(dataMaid)
library(gtsummary)
library(flextable)
library(Boruta)
library(ggstatsplot)
library(caret)
library(leaps)
library(splines)
library(boot)
library(pROC)

db_raw <- read_csv(here::here('data/database.csv')) %>%
  mutate(across(c(nps_index, sex, gfap_046, clancy, rigby, stat, preterm), as.factor))
```

Data Check

```
check(db_raw)
```

Descriptives

```
db_raw %>%

  mutate(nps_index = recode_factor(nps_index,
                                   `0` = "Non-impaired", `1` = "Impaired")) %>%
  select(-id) %>%

  tbl_summary
```

Variable importance

Boruta algorithm aims to identify which are all the relevant predictors that impact the outcome of interest (in our case, the belonging to the impaired not impaired NDI group). It implements a random forest on an augmented set of covariates. Additional covariates, called shadow variables, are copies of the original ones obtained by permuting the observations and thus removing the eventual association with the outcome. For each explanatory variable, an importance measure is computed, i.e., the Z-score, which is the average improvement in the predictive performance of the random forest with the considered explanatory variable divided by its standard deviation. The obtained important predictors are those that show a Z-score higher than the one observed for the variable with the maximum Z-score among the shadow variables. The procedure is repeated until an importance measure is assigned to each predictor or until the maximum number of random forests is reached.

```
## Boruta

db_boruta <- db_raw %>% select(-id, -gfap_max_log, -gfap_046, -hypothermia_min_temp)

set.seed(3011)

boruta.gfap_train <- Boruta(nps_index ~ ., data = db_boruta)

boruta.gfap_train

boruta.gfap <- TentativeRoughFix(boruta.gfap_train)

boruta.gfap

#tiff(filename = "Figure_2.tif", width=6, height=4, units="in", res = 1200, compression = "lz
w")
plot(boruta.gfap, xlab = "", las = 3, cex.axis = 0.8,
     whichShadow = c(FALSE, FALSE, FALSE),
     pars = par(mar=c(9.5,4,2,1)))
#dev.off()

gfap_df <- attStats(boruta.gfap) %>% relocate(decision) %>% arrange(decision, desc(meanImp))
kbl(gfap_df, caption = "Importance") %>% kable_paper("hover", full_width = FALSE)
```

Correlation matrix

```
ggstatsplot::ggcorrmat(
  data = db_boruta,
  type = 'np' #non-parametric
)
```

Best subset selection

```
 # non collinear variables were selected

db_models <- db_boruta %>% select(-surgery_time, -age,
                                  -hypothermia, -rewarming) %>%

  mutate(across(c(rigby, clancy, stat), as.numeric))

models <- regsubsets(nps_index ~ ., data = db_models, nvmax = 500, method = "exhaustive")

summary(models)

plot(models, scale="bic")

res.sum <- summary(models)

best_models <- data.frame(
  Adj.R2 = which.max(res.sum$adjr2),
  CP = which.min(res.sum$cp),
  BIC = which.min(res.sum$bic)
)

best_models
```

Spline selection

```
knots = purrr::set_names(c(0.2, 0.3, 0.4, 0.5, 0.6, 0.7, 0.8, 1, 2))

glm_knot <- function(k) {
  glm.spline <- glm(nps_index ~ bs(gfap_max, knots = k) + icu_days + cpb_time + clancy + weig
ht + min_temp,
                    data =  db_boruta,
                    family = binomial)

  cv.glm(db_boruta, glm.spline)$delta[[1]]

}

purrr::map_dbl(knots, glm_knot)



knots_2 <- purrr::set_names(c(0.41, 0.42, 0.43, 0.44, 0.45, 0.46, 0.48, 0.49))

purrr::map_dbl(knots_2, glm_knot)

which.min(purrr::map_dbl(knots_2, glm_knot)) # min 0.49
```

Logistic regression

```
glm.spline <- glm(nps_index ~ bs(gfap_max, knots = .49) + icu_days + cpb_time + clancy + weig
ht + min_temp,
                  data =  db_boruta,
                  family = binomial)

summary(glm.spline)

glm.spline_probs <- predict(glm.spline, type = 'response')

glm.spline_pred <- rep("0", 38)
glm.spline_pred[glm.spline_probs>0.5] = "1"

table(glm.spline_pred, db_boruta$nps_index)

mean(glm.spline_pred == db_boruta$nps_index)

ggplot(db_boruta, aes(x=gfap_max, y=log(glm.spline_probs))) +

  geom_point() +

  geom_segment(aes(x=0, y=-6.5, xend = 2, yend = 0, colour = "segment"))+

  geom_segment(aes(x = 2, y = 0, xend = 8, yend = 0, colour = "segment")) +

  geom_smooth(se = FALSE, method = "gam")


# unnested model (null model)
glm.spline_un <- glm(nps_index ~ gfap_max + icu_days + cpb_time + clancy + weight + min_temp,
                     data =  db_boruta,
                     family = binomial)

summary(glm.spline_un)

anova(glm.spline, glm.spline_un, test = "Chisq")
```

ROC

```
roc <- pROC::roc(db_boruta$nps_index ~ glm.spline_probs,
                 data = db_boruta,
                 ci = TRUE,
                 plot = TRUE)
```

Descriptives NDI and GFAP

```
db_boruta %>% tbl_summary (by = nps_index)

db_boruta %>% mutate(gfap_049 = ifelse(gfap_max > 0.49, 1, 0)) %>%
  tbl_summary (by = gfap_049)
```
