# Supplementary material for: Perioperative Glial Fibrillary Acidic Protein Is Associated with Long-Term Neurodevelopment Outcome of Infants with Congenital Heart Disease
Source: Children (Basel). 2021 Jul 29;8(8):655. doi: 10.3390/children8080655 (PMC8391328; doi:10.3390/children8080655)
Supplement: Supplementary file 1 [file children-08-00655-s001.zip › FIle S2_R packages used.pdf]

R packages used

- base (R Core Team 2021a)
- boot (Davison and Hinkley 1997)
- Boruta (Kursa and Rudnicki 2010)
- caret (Kuhn 2020)
- dataMaid (Petersen and Ekstrøm 2019)
- flextable (Gohel 2021)
- ggstatsplot (Patil 2021)
- gtsummary (Sjoberg et al. 2021)
- here (Müller 2020)
- kableExtra (Zhu 2021)
- leaps (Fortran code by Alan Miller 2020)
- pROC (Robin et al. 2011)
- purrr (Henry and Wickham 2020)
- randomForestSRC (Ishwaran et al. 2008)
- readr (Wickham and Hester 2020)
- rms (Harrell Jr 2021)
- splines (R Core Team 2021b)
- tidyverse (Wickham et al. 2019)

References

Davison, A. C., and D. V. Hinkley. 1997. *Bootstrap Methods and Their Applications*. Cambridge: Cambridge University Press. http://statwww.epfl.ch/davison/BMA/ (http://statwww.epfl.ch/davison/BMA/).

Fortran code by Alan Miller, Thomas Lumley based on. 2020. *Leaps: Regression Subset Selection*. https://CRAN.R-project.org/package=leaps (https://CRAN.R-project.org/package=leaps).

Gohel, David. 2021. *Flextable: Functions for Tabular Reporting*. https://CRAN.R-project.org/package=flextable (https://CRAN.R-project.org/package=flextable).

Harrell Jr, Frank E. 2021. *Rms: Regression Modeling Strategies*. https://CRAN.R-project.org/package=rms (https://CRAN.R-project.org/package=rms).

Henry, Lionel, and Hadley Wickham. 2020. *Purrr: Functional Programming Tools*. https://CRAN.R-project.org/package=purrr (https://CRAN.R-project.org/package=purrr).

Ishwaran, H., U. B. Kogalur, E. H. Blackstone, and M. S. Lauer. 2008. “Random Survival Forests.” *Ann. Appl. Statist.* 2 (3): 841–60. https://arXiv.org/abs/0811.1645v1 (https://arXiv.org/abs/0811.1645v1).

Kuhn, Max. 2020. *Caret: Classification and Regression Training*. https://CRAN.R-project.org/package=caret (https://CRAN.R-project.org/package=caret).

Kursa, Miron B., and Witold R. Rudnicki. 2010. “Feature Selection with the Boruta Package.” *Journal of Statistical Software* 36 (11): 1–13. http://www.jstatsoft.org/v36/i11/ (http://www.jstatsoft.org/v36/i11/).

Müller, Kirill. 2020. *Here: A Simpler Way to Find Your Files*. https://CRAN.R-project.org/package=here (https://CRAN.R-project.org/package=here).

Patil, Indrajeet. 2021. “Visualizations with Statistical Details: The ’Ggstatsplot’ Approach.” *PsyArxiv*. https://doi.org/10.31234/osf.io/p7mku (https://doi.org/10.31234/osf.io/p7mku).

Petersen, Anne Helby, and Claus Thorn Ekstrøm. 2019. “dataMaid: Your Assistant for Documenting Supervised Data Quality Screening in R.” *Journal of Statistical Software* 90 (6): 1–38. https://doi.org/10.18637/jss.v090.i06 (https://doi.org/10.18637/jss.v090.i06).

R Core Team. 2021a. *R: A Language and Environment for Statistical Computing*. Vienna, Austria: R Foundation for Statistical Computing. https://www.R-project.org/ (https://www.R-project.org/).

———. 2021b. *R: A Language and Environment for Statistical Computing*. Vienna, Austria: R Foundation for Statistical Computing. https://www.R-project.org/ (https://www.R-project.org/).

Robin, Xavier, Natacha Turck, Alexandre Hainard, Natalia Tiberti, Frédérique Lisacek, Jean-Charles Sanchez, and Markus Müller. 2011. "pROC: An Open-Source Package for r and s+ to Analyze and Compare ROC Curves." *BMC Bioinformatics* 12: 77.

Sjoberg, Daniel D., Michael Curry, Margie Hannum, Joseph Larmarange, Karissa Whiting, and Emily C. Zabor. 2021. *Gtsummary: Presentation-Ready Data Summary and Analytic Result Tables*. https://CRAN.R-project.org/package=gtsummary (https://CRAN.R-project.org/package=gtsummary).

Wickham, Hadley, Mara Averick, Jennifer Bryan, Winston Chang, Lucy D'Agostino McGowan, Romain François, Garrett Grolemund, et al. 2019. "Welcome to the tidyverse." *Journal of Open Source Software* 4 (43): 1686. https://doi.org/10.21105/joss.01686 (https://doi.org/10.21105/joss.01686).

Wickham, Hadley, and Jim Hester. 2020. *Readr: Read Rectangular Text Data*. https://CRAN.R-project.org/package=readr (https://CRAN.R-project.org/package=readr).

Zhu, Hao. 2021. *kableExtra: Construct Complex Table with 'Kable' and Pipe Syntax*. https://CRAN.R-project.org/package=kableExtra (https://CRAN.R-project.org/package=kableExtra).
