# Supplementary material for: Perioperative Glial Fibrillary Acidic Protein Is Associated with Long-Term Neurodevelopment Outcome of Infants with Congenital Heart Disease
Source: Children (Basel). 2021 Jul 29;8(8):655. doi: 10.3390/children8080655 (PMC8391328; doi:10.3390/children8080655)
Supplement: Supplementary file 1 [file children-08-00655-s001.zip › Table S1.docx]

**Table S1.** NDI and GFAP groups characteristics.

| **Characteristic** | **GFAP ≤ 0.49 ng/ml**  **N = 11^1^** | **GFAP > 0.49 ng/ml**  **N = 27^1^** | **NDI normal**  **N = 16^1^** | **NDI impaired**  **N = 22^1^** |
| --- | --- | --- | --- | --- |
| **NDI Index** |  |  |  |  |
| Normal | 9 (82%) | 7 (26%) |  |  |
| Impaired | 2 (18%) | 20 (74%) |  |  |
| **Sex** |  |  |  |  |
| Male | 4 (36%) | 18 (67%) | 7 (44%) | 15 (68%) |
| Female | 7 (64%) | 9 (33%) | 9 (56%) | 7 (32%) |
| **Age** | 35 (16, 44) | 2 (0, 6) | 14 (4, 42) | 4 (0, 8) |
| **Weight** | 14.1 (9.7, 15.5) | 4.2 (3.3, 6.8) | 8.4 (5.2, 14.7) | 4.8 (3.3, 7.5) |
| **Surgery duration (min)** | 220 (175, 242) | 250 (210, 298) | 248 (188, 255) | 245 (201, 304) |
| **CPB duration (min)** | 74 (60, 108) | 142 (114, 162) | 100 (66, 133) | 142 (111, 158) |
| **Hypothermia duration (min)** | 46 (30, 60) | 80 (70, 101) | 60 (38, 82) | 80 (70, 90) |
| **CPB minimum temperature (°C)** | 32.9 (32.4, 34.0) | 30.7 (27.5, 32.0) | 32.4 (32.0, 33.6) | 29.9 (25.5, 32.0) |
| **Rewarming duration (min)** | 20 (15, 26) | 34 (30, 40) | 26 (18, 31) | 33 (30, 58) |
| **GFAP peak (ng/ml)** | 0.26 (0.10, 0.41) | 1.34 (0.88, 1.91) | 0.44 (0.30, 1.00) | 1.32 (0.73, 1.92) |
| **Clancy** |  |  |  |  |
| 1 | 5 (45%) | 20 (74%) | 11 (69%) | 14 (64%) |
| 2 | 0 (0%) | 2 (7.4%) | 0 (0%) | 2 (9.1%) |
| 3 | 4 (36%) | 0 (0%) | 2 (12%) | 2 (9.1%) |
| 4 | 2 (18%) | 5 (19%) | 3 (19%) | 4 (18%) |
| **Rigby** |  |  |  |  |
| 1 | 5 (45%) | 14 (52%) | 8 (50%) | 11 (50%) |
| 2 | 2 (18%) | 6 (22%) | 3 (19%) | 5 (23%) |
| 3 | 4 (36%) | 7 (26%) | 5 (31%) | 6 (27%) |
| **STAT** |  |  |  |  |
| 1 | 1 (9.1%) | 4 (15%) | 2 (12%) | 3 (14%) |
| 2 | 8 (73%) | 7 (26%) | 8 (50%) | 7 (32%) |
| 3 | 1 (9.1%) | 6 (22%) | 3 (19%) | 4 (18%) |
| 4 | 1 (9.1%) | 7 (26%) | 3 (19%) | 5 (23%) |
| 5 | 0 (0%) | 3 (11%) | 0 (0%) | 3 (14%) |
| **Preterm** |  |  |  |  |
| No | 11 (100%) | 21 (78%) | 14 (88%) | 18 (82%) |
| Yes | 0 (0%) | 6 (22%) | 2 (12%) | 4 (18%) |
| **ICU (d)** | 5 (2, 6) | 5 (2, 7) | 5 (3, 5) | 5 (2, 8) |
| **Length of Stay (d)** | 12 (8, 16) | 12 (8, 16) | 12 (8, 14) | 12 (8, 17) |
| ^1^n (%); Median (IQR) | | |  |  |
